# Agonist antibody to MuSK protects mice from MuSK myasthenia gravis

**DOI:** 10.1073/pnas.2408324121

**Published:** 2024-09-17

**Authors:** Julien Oury, Begona Gamallo-Lana, Leah Santana, Christophe Steyaert, Dana L. E. Vergoossen, Adam C. Mar, Bernhardt Vankerckhoven, Karen Silence, Roeland Vanhauwaert, Maartje G. Huijbers, Steven J. Burden

**Affiliations:** ^a^Helen L. and Martin S. Kimmel Center for Biology and Medicine at the Skirball Institute, New York University Medical School, New York, NY 10016; ^b^Department of Neuroscience and Physiology, Neuroscience Institute, New York University School of Medicine, New York, NY 10016; ^c^argenx, Zwijnaarde 9052, Belgium; ^d^Department of Human Genetics, Leiden University Medical Centre, Leiden 2300 RC, The Netherlands; ^e^Department of Neurology, Leiden University Medical Centre, Leiden 2300 RC, The Netherlands

**Keywords:** autoimmune disease, myasthenia gravis, therapeutic antibody, neuromuscular, synapse

## Abstract

Myasthenia gravis (MG) is a chronic and severe disease of the skeletal neuromuscular junction (NMJ) in which the effects of neurotransmitters are attenuated, leading to muscle weakness. In the most common forms of autoimmune MG, antibodies attack components of the postsynaptic membrane, including the acetylcholine receptor (AChR) or muscle-specific kinase (MuSK). MuSK, a master regulator of NMJ development, associates with the low-density lipoprotein-related receptor 4 (Lrp4) to form the signaling receptor for neuronal Agrin, a nerve-derived synaptic organizer. Pathogenic antibodies to MuSK interfere with binding between MuSK and Lrp4, inhibiting the differentiation and maintenance of the NMJ. MuSK MG can be debilitating and refractory to treatments that are effective for AChR MG. We show here that recombinant antibodies, derived from MuSK MG patients, cause severe neuromuscular disease in mice. The disease can be prevented by a MuSK agonist antibody, presented either prophylactically or after disease onset. These findings suggest a therapeutic alternative to generalized immunosuppression for treating MuSK MG by selectively and directly targeting the disease mechanism.

## Significance

Autoimmune diseases are typically managed by generalized immunosuppression. Despite their benefits, these treatments render patients vulnerable to infection and dampen antibody-mediated surveillance mechanisms. Here, we provide evidence for a therapeutic strategy to treat an autoimmune disease by selectively targeting the disease mechanism. Autoantibodies to muscle-specific kinase (MuSK) cause myasthenia gravis (MG). MuSK MG is a debilitating disease, causing relapsing phases of muscle weakness and fatigue, often requiring ventilation. We show that passive transfer of autoantibodies, derived from MuSK MG patients, causes severe neuromuscular deficits in mice, which are reversed after disease onset by a MuSK agonist antibody. These findings suggest a therapeutic alternative to generalized immunosuppression for treating MuSK MG by selectively and directly targeting the disease mechanism.

## Muscle-SpecificKinase (MuSK), A Key Player for Forming and Maintaining Neuromuscular Synapses.

The formation and maintenance of neuromuscular synapses requires the coordinated action of key signaling molecules expressed by motor neurons and skeletal muscle (1–3). Agrin, produced by motor neurons, binds low-density lipoprotein-related receptor 4 (Lrp4) in muscle, increasing association between Lrp4 and MuSK and stimulating MuSK tyrosine phosphorylation (4–10). MuSK serves as a master regulator of synaptic differentiation, as phosphorylated MuSK stimulates the clustering and anchoring of postsynaptic proteins, focally enhances transcription of genes encoding synaptic proteins in subsynaptic muscle nuclei and clusters Lrp4, which signals back to motor neurons to stimulate presynaptic differentiation (2, 3, 11, 12). Importantly, MuSK-dependent signaling is required not only for forming neuromuscular synapses during development but also for maintaining neuromuscular synapses throughout adult life (11).

## Autoantibodies to MuSK Impair Synaptic Differentiation.

Disruptions in this pathway for building and maintaining neuromuscular synapses are responsible for neuromuscular disease (11). Mutations in genes that govern the assembly and maintenance of neuromuscular synapses, or mediate synaptic transmission, are responsible for congenital myasthenia, whereas autoantibodies to key postsynaptic proteins, such as AChRs and MuSK, are responsible for autoimmune myasthenia gravis (MG) (13, 14). Most MG patients carry autoantibodies to AChRs, but ~8% of MG patients instead carry autoantibodies to MuSK, which cause relapsing phases of muscle weakness and fatigue (14–17).

Autoantibodies that cause MuSK MG are largely of the IgG4 subclass, which are functionally monovalent and fail to bind complement (18). The predominant target for these antibodies is the first Ig-like domain in MuSK, which is required for the association between Lrp4 and MuSK (10, 19). Consequently, serum IgG4 autoantibodies to MuSK inhibit binding between Lrp4 and MuSK, impairing Agrin-stimulated MuSK phosphorylation and the signaling pathways required to form and maintain neuromuscular synapses (20, 21).

MuSK MG can be debilitating as ~30% of patients require ventilation at some point in their lifetime (22). Moreover, MuSK MG is difficult to manage, as treatments that are effective for AChR MG, such as inhibition of complement and acetylcholinesterase, are ineffective and can be harmful for MuSK MG patients (23). As such, MuSK MG is managed by traditional treatments, including plasmapheresis and generalized immunosuppression, which present their own risks. Here, using a mouse model of MuSK MG, we show that MuSK agonist antibodies, provided before or after symptom onset, can prevent disease caused by pathogenic MuSK antagonist antibodies. These results suggest a therapeutic strategy for MuSK MG that directly and selectively targets the disease mechanism.

## Results

### A Mouse Model of MuSK MG.

We studied a passive transfer mouse model of MuSK MG, induced by injecting wildtype C57BL/6 mice with two different recombinant, monovalent pathogenic antibodies to MuSK, derived from a MuSK MG patient (17, 24–26).These recombinant antibodies, like most antibodies in sera from MuSK MG patients, are directed against the first Ig-like domain in MuSK and inhibit Agrin-stimulated MuSK phosphorylation (17, 24, 27). Because most autoantibodies from MuSK MG patients are IgG4, which undergo Fab arm exchange and are functionally monovalent, we used a one-armed, recombinant 3F6C or 3B5 antibody to MuSK, which has a single Fab arm that binds MuSK (17, 24). As such, these functionally monovalent antibodies do not force dimerize and activate MuSK but rather inhibit the ability of Agrin to stimulate MuSK phosphorylation (17).

**Fig. 1.**
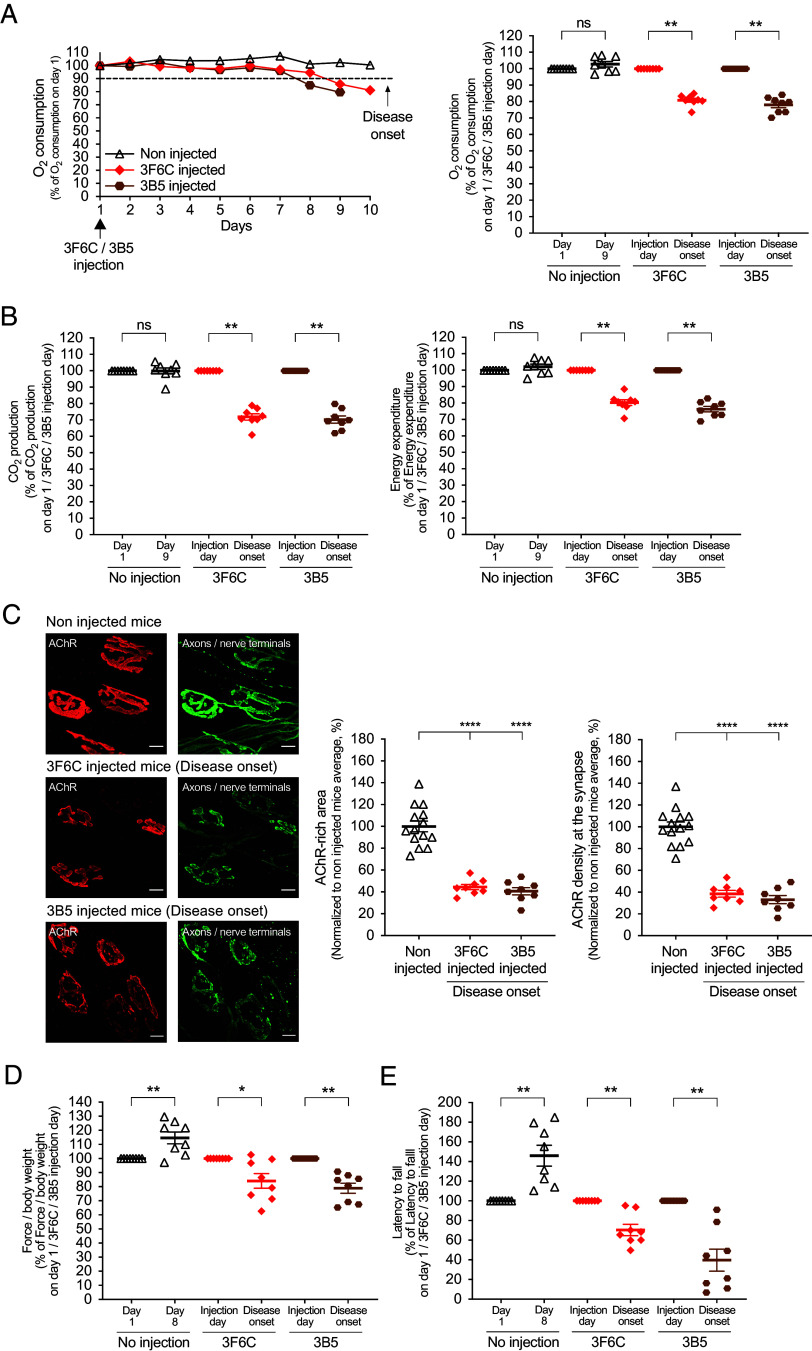
Monovalent 3F6C and 3B5 antibodies cause neuromuscular disease. (*A*) Mice reached disease onset when O_2_ consumption was reduced by 10% on two consecutive days, as shown for a single control mouse and a single mouse injected with 5 mg/kg either 3F6C or 3B5 (*Left*). The scatter plot (*Right*) shows the mean ± SEM levels of O_2_ consumption at disease onset (n = 8 control mice and eight mice injected with either 3F6C or 3B5). The mean values on day 1 were set as 100%. (*B*) At disease onset, CO_2_ production and energy expenditure were reduced by 20 to 30%. (*C*) At disease onset, synaptic size and AChR density were reduced by 60 to 70% (AChRs, red; axons and nerve terminals, green). (Scale bar, 10 m (micrometers)) The scatter plot shows the mean ± SEM values for >50 synapses/mouse in ≥8 mice. The mean values for control mice were set at 100%. (*D, E*) At disease onset, grip strength and performance on a rotarod were reduced (n = 8 mice injected with 3F6C or 3B5 and n = 8 noninjected control mice). A Mann–Whitney test was used in panels *A*, *B*, *D*, and *E*.

Mice received a single, intraperitoneal injection of functionally monovalent 3F6C or 3B5. We monitored the mice continuously in metabolic chambers and found that mice treated with 3F6C or 3B5 began to develop signs of neuromuscular disease 1 wk later, when O_2_ consumption began to diminish (Fig. 1 *A* and *B*). By 7 to 9 d after injection with 3F6C or 3B5 (5 mg/kg), O_2_ consumption was reduced by >10% in all (8/8) mice, which we defined as disease onset (Fig. 1*A*). At this time, CO_2_ production and energy expenditure were similarly reduced (Fig. 1*B*). In addition, synaptic size and synaptic AChR density were reduced by ~60% (Fig. 1*C*), and grip strength and performance on an accelerating rotarod were diminished by 20% and 50%, respectively (Fig. 1*D*). Control, wildtype mice, which were not injected with a pathogenic antibody but housed in the same metabolic chambers, showed no decline in motor performance (Fig. 1*D*). Two to three weeks after injection of 3F6C or 3B5, the experiment was terminated, as O_2_ consumption in all of the mice was reduced by >50%, which inevitably foreshadowed death within a day and defined disease endpoint.

### Reversing MuSK Inhibition in Cell Culture.

**Fig. 2.**
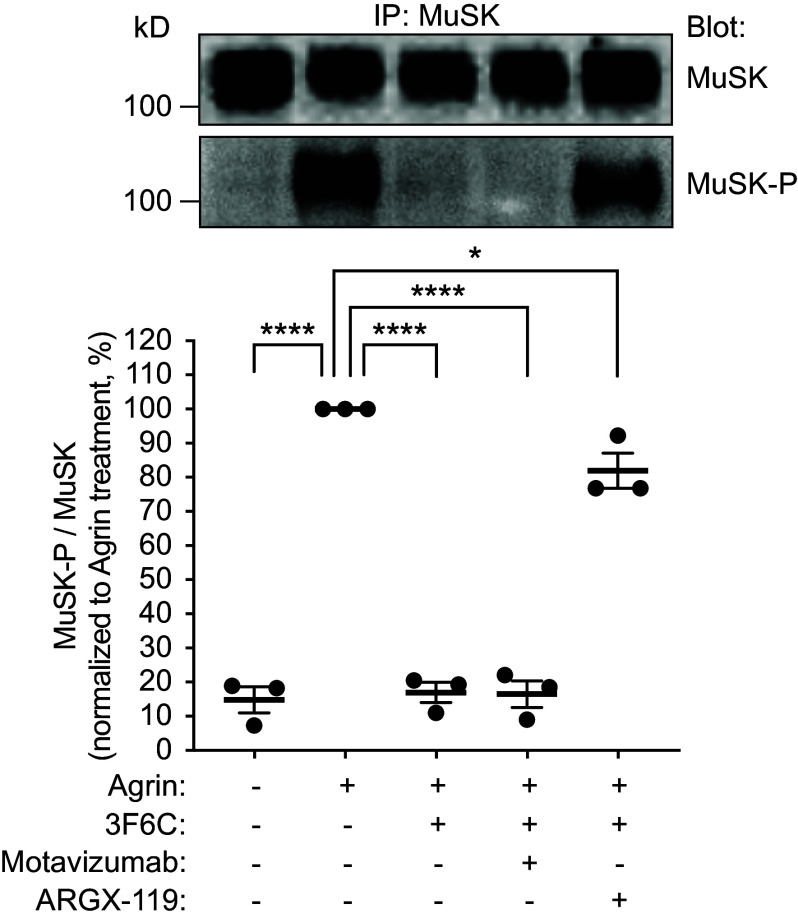
ARGX-119 reverses inhibition of Agrin-stimulated MuSK phosphorylation caused by 3F6C in cultures myotubes. C2C12 myotubes were treated with neuronal Agrin (1 nM), neuronal Agrin and monovalent 3F6C (10 nM), or neuronal Agrin and one-armed 3F6C, followed 30 min later by either motavizumab (10 nM) or ARGX-119 (10 nM). MuSK was immunoprecipitated, and Western blots were probed with antibodies to MuSK or p-Tyr (4G10) (28). MuSK phosphorylation was normalized to MuSK expression. 3F6C reduced Agrin-stimulated MuSK phosphorylation by 10-fold; subsequent treatment with ARGX-119 increased MuSK phosphorylation by eightfold. The scatter plot shows the mean (±SEM) levels of Agrin-stimulated MuSK phosphorylation, normalized to the level of MuSK expression, from three separate experiments.

Because autoantibodies to MuSK inhibit MuSK phosphorylation, we assessed the viability of a MuSK agonist antibody to overcome disease. We first tested whether a MuSK agonist antibody, ARGX-119, could overcome the inhibition of Agrin-stimulated MuSK phosphorylation, caused by 3F6C in cultured C2C12 myotubes (27). ARGX-119, like MuSK agonist antibodies described previously (28), recognizes the Fz-like domain in human and mouse MuSK and stimulates MuSK phosphorylation in cultured myotubes (29). Unlike MuSK agonist antibodies that target the essential first Ig-like domain in MuSK, ARGX-119 targets the dispensable Fz-like domain in MuSK (30–32). Like other MuSK agonist antibodies that bind the Fz-like domain, chronic dosing with ARGX-119 caused no ill-effects in mice (28, 29, 33, 34). The antibody was engineered on a hIgG1 backbone with mutations (L234A, L235A), which diminished effector function (29, 35). Myotubes were treated with either Agrin alone, Agrin and 3F6C, or Agrin and 3F6C, followed 30 min later with ARGX-119. 3F6C prevented Agrin-stimulated MuSK phosphorylation, which was restored to near normal levels (~80% of Agrin alone) by subsequent treatment with ARGX-119 (Fig. 2). These findings demonstrated that ARGX-119 was capable of overcoming and reversing inhibition of MuSK phosphorylation caused by 3F6C, suggesting that ARGX-119 had the potential to reverse the pathogenic effects of 3F6C in vivo.

### Prophylactic Protection.

**Fig. 3.**
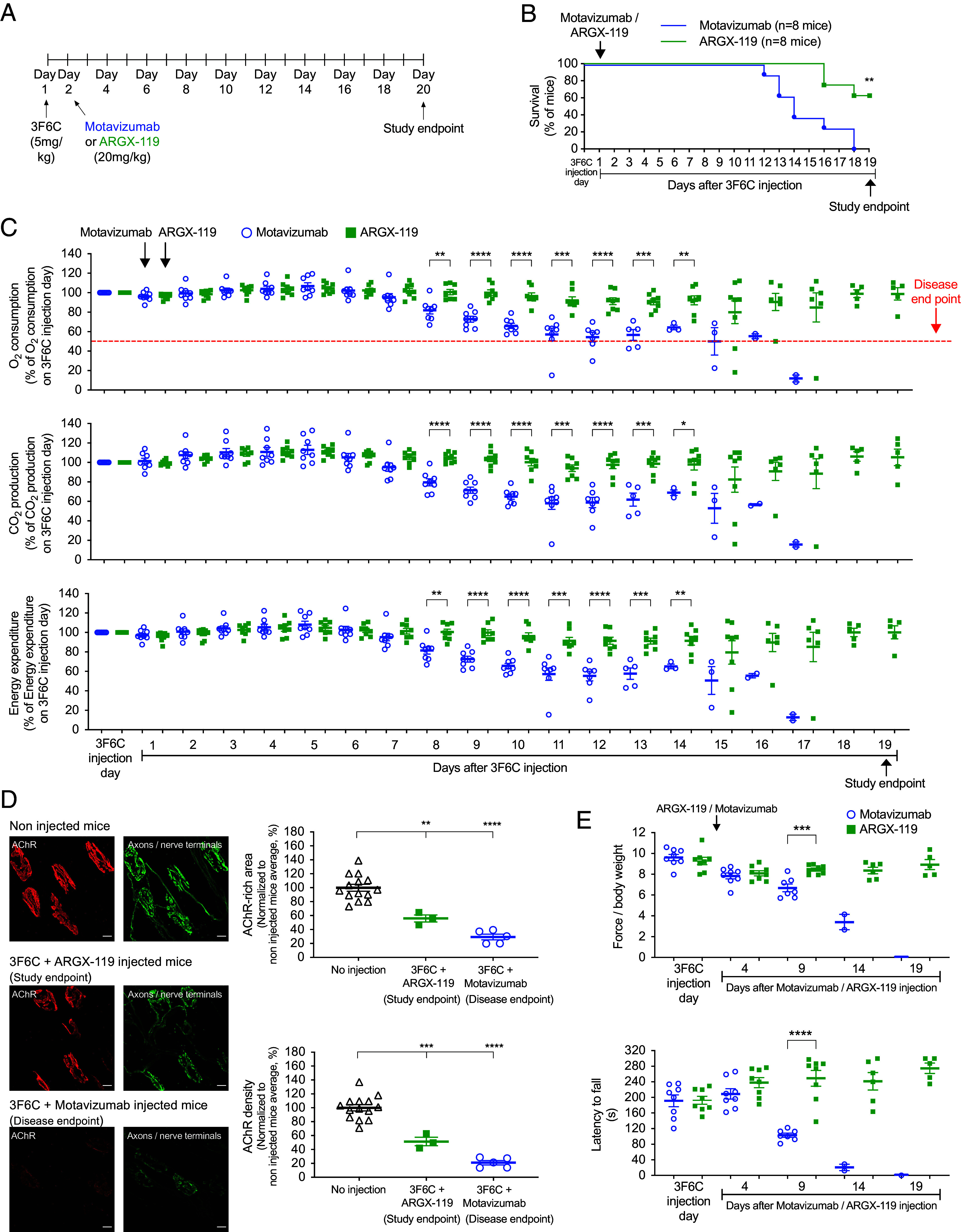
Prophylactic treatment with ARGX-119 protects mice from neuromuscular disease caused by 3F6C. (*A*) Mice were injected with 3F6C and 1 d later with either motavizumab or ARGX-119. (*B*) All eight motavizumab-treated mice reached disease endpoint. 5/8 ARGX-119-treated mice survived to study endpoint. Log-rank (Mantel-Cox) test (**P < 0.005). (*C*) Within a week after motavizumab injection, O_2_ consumption, CO_2_ production, and lower energy expenditure were diminished, and all eight mice reached disease endpoint by 2 wk. Most (5/8) ARGX-119-treated mice failed to develop signs of disease. (*D*) AChRs (red) motor axons and nerve terminals (green) were stained at disease endpoint for motavizumab-treated mice and at study endpoint for surviving ARGX-119-treated mice. (Scale bar, 10 m (micrometers)) Synaptic size and AChR density were reduced by 77% and 70%, respectively, at disease endpoint in motavizumab-treated mice and by 50% at study endpoint in ARGX-treated mice. (*E*) Motor performance declined in motavizumab-treated mice, whereas ARGX-119-treatment fully protected mice from the debilitating effects of 3F6C.

We first sought to determine whether prophylactic treatment with ARGX-119 could prevent disease in mice treated with 3F6C. ARGX-119, like the X17 MuSK agonist antibody described previously (28), bound MuSK at neuromuscular synapses in vivo in a dose-dependent and saturable manner, had a 12- to 14-d half-life in blood, and caused no ill effects in chronically dosed female and male mice (29). One day after injection of 3F6C, but prior to signs of disease, we injected mice with a near-saturating dose of ARGX-119 (20 mg/kg) or an isotype-matched negative control antibody, motavizumab (20 mg/kg) (Fig. 3*A*).

Mice injected with 3F6C, followed a day later by motavizumab, showed the same time course and signs of disease as mice that received only 3F6C (Figs. 1 and 3 *B*–*E*). O_2_ consumption in all (8/8) of the motavizumab-treated mice was reduced by >50% within 2 to 3 wk after 3F6C treatment, when the experiment was terminated (Fig. 3 *B* and *C*). In contrast, mice that were treated with ARGX-119 1 d after injection of 3F6C were largely protected from disease, as assessed by survival, O_2_ consumption, CO_2_ production, and energy expenditure, for nearly 3 wk when the study was ended (Fig. 3 *B* and *C*). Over 60% (5/8) of the ARGX119-treated mice survived until the study endpoint (Fig. 3*B*). Moreover, ARGX-119 partially protected neuromuscular synapses from deterioration, as assessed by synaptic size and density of synaptic AChRs (Figs. 1*C* and 3*D*). Importantly, ARGX-119 fully prevented the decline in motor behavior, as measured by grip strength and performance on a rotarod (Figs. 1*D* and 3*E*). These findings demonstrated that ARGX-119, presented prophylactically before disease symptoms became evident, prevented the severe motor deficits and lethality caused by a patient-derived, pathogenic antibody to MuSK.

### Reversal after Disease Onset.

**Fig. 4.**
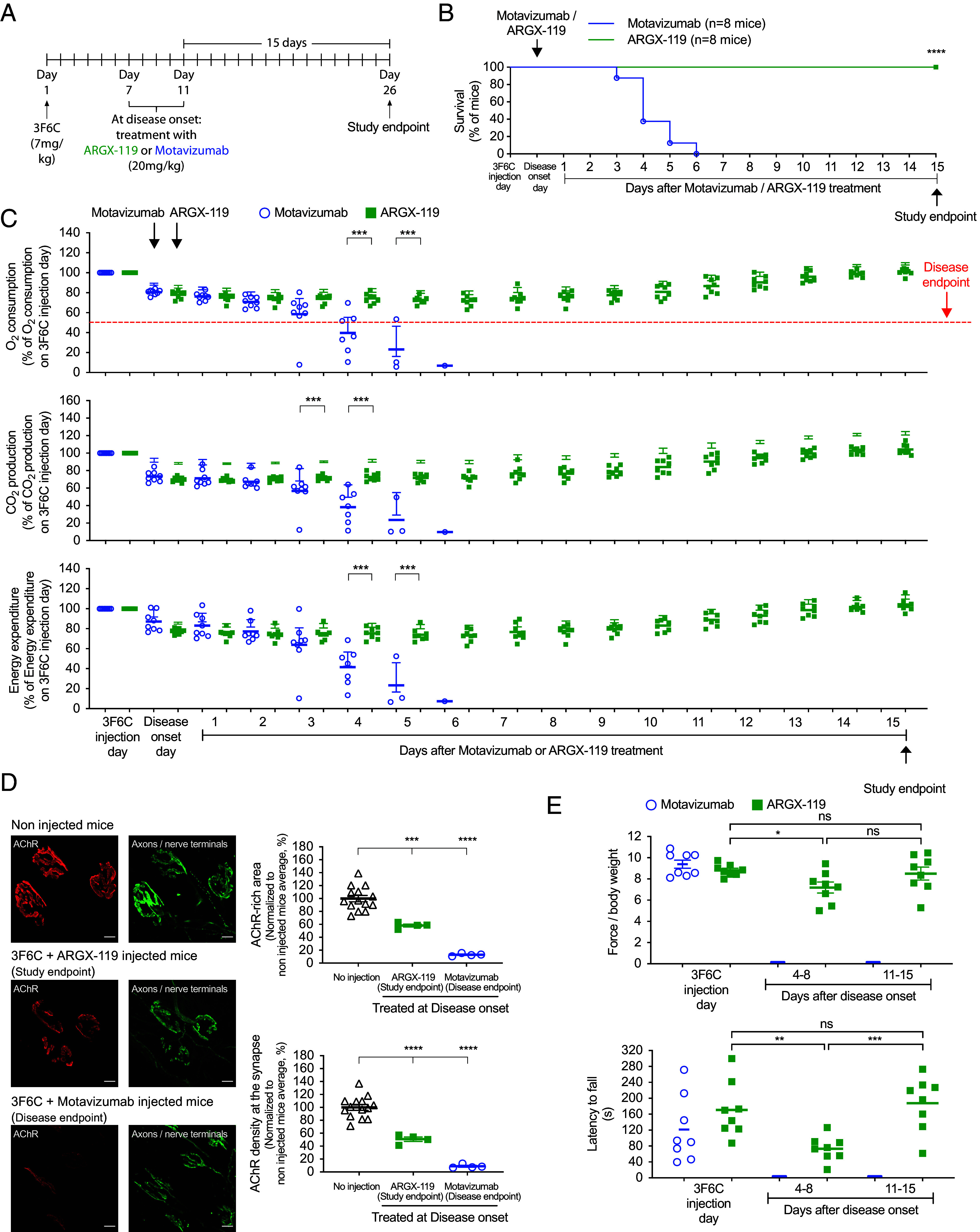
ARGX-119 rescued mice from neuromuscular disease caused by monovalent 3F6C. (*A*) Mice were injected with 3F6C and either motavizumab or ARGX-119 at disease onset. (*B*) ARGX-119 rescued mice from lethality: All eight ARGX-119-injected mice survived to study endpoint, whereas all eight motavizumab-treated mice reached disease endpoint within 5 d after disease onset. Plots show the percentage of surviving mice. Log-rank (Mantel-Cox) test (****P < 0.0005; graph). (*C*) ARGX-119 reversed the decline in O_2_ consumption, CO_2_ production, and energy expenditure in all eight mice. In contrast, all eight mice treated with motavizumab continued to decline and reached disease endpoint 5 d after disease onset. (*D*) AChRs (red) and motor axons and nerve terminals (green) were visualized at disease endpoint for motavizumab-treated mice and at study endpoint for ARGX-119-treated mice. (Scale bar, 10 m (micrometers)) Synaptic size and AChR density were reduced by 88% and 90%, respectively, in mice injected with motavizumab. Synaptic deterioration was partially protected by ARGX-119, as synaptic size and synaptic AChR density were reduced by 41% and 55%, respectively. (*E*) Motavizumab-treated mice reached disease endpoint before motor performance could be assessed. In contrast, ARGX-119-treatment halted the decline in grip strength and improved rotarod performance.

We next sought to determine whether ARGX-119, delivered after disease onset, could reverse symptoms of disease caused by 3F6C. We continuously recorded disease progression of mice injected with 3F6C by monitoring individual mice in metabolic chambers, and we initiated treatment with ARGX-119 or motavizumab at disease onset, when O_2_ consumption was reduced by >10% for two consecutive days (Fig. 4*A*). At this time, a week after treatment with the pathogenic antibody, the structure of neuromuscular synapses and motor performance had deteriorated substantially (Fig. 1*D*).

Mice that were treated with 3F6C (7 mg/kg), followed by motavizumab after disease onset, failed to recover from neuromuscular disease: The respiratory deficits continued to wane in all (8/8) of the mice, and the experiment was terminated when O_2_ consumption dropped by >50% (Fig. 4 *B* and *C*). At disease endpoint, synaptic size and the density of synaptic AChRs were reduced by 10-fold (Fig. 4*D*).

In contrast, treatment with ARGX-119, initiated after disease onset, led to a gradual recovery from the respiratory deficits caused by 3F6C (Fig. 4 *B* and *C*), and all (8/8) of the ARGX-119-treated mice survived until the study was ended 15 d later (Fig. 4*B*). Further, ARGX-119 halted the prior decline in synaptic size and AChR density, evident at disease onset (55% and 60%, respectively) (Figs. 1*C* and 4*D*). Importantly, 2 wk after treatment with ARGX-119, motor performance returned to normal, as the grip strength and rotarod performance of the ARGX-119-treated mice were indistinguishable from wild type control mice (Figs. 1*D* and 4*E*).

**Fig. 5.**
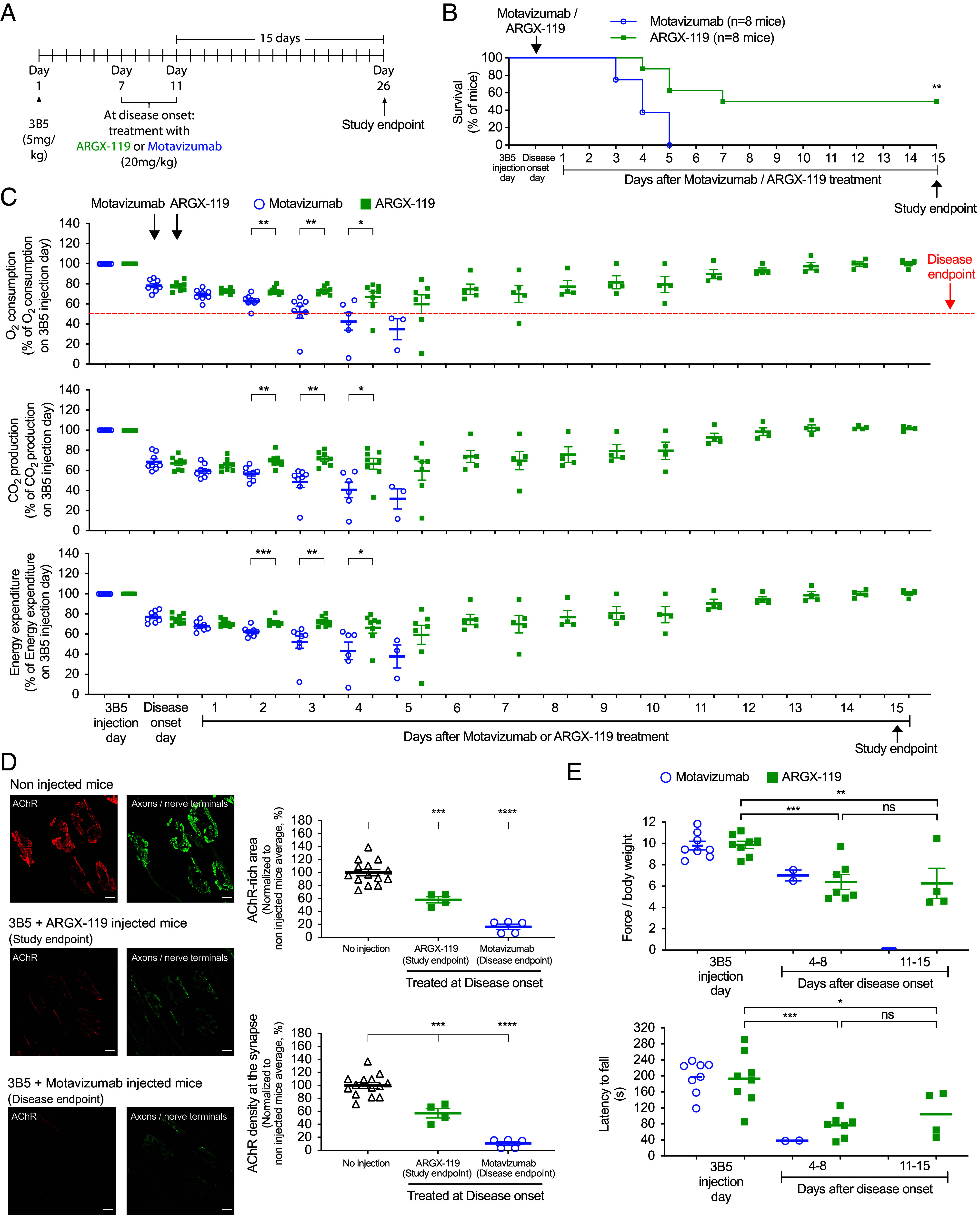
ARGX-119 rescued mice from neuromuscular disease caused by monovalent 3B5. (*A*) Mice were injected with 3B5 and either motavizumab or ARGX-119 at disease onset. (*B*) ARGX-119 rescued mice from lethality: half (4/8) of the ARGX-119-treated mice survived to study endpoint, whereas all eight motavizumab-treated mice reached disease endpoint within 5 d after disease onset. Plots show the percentage of surviving mice. Log-rank (Mantel-Cox) test (**P < 0.005). (*C*) ARGX-119 reversed the decline in O_2_ consumption, CO_2_ production, and energy expenditure in half (4/8) of the mice. In contrast, all eight motavizumab-treated mice continued to decline and reached disease endpoint 5 d after disease onset. (*D*) AChRs (red) and motor axons and nerve terminals (green) were visualized at disease endpoint for motavizumab-treated mice and at study endpoint for ARGX-119-treated and untreated mice. (Scale bar, 10 m (micrometers)) Synaptic size and AChR density were reduced by 84% and 90%, respectively, in motavizumab-treated mice and by 40% in ARGX-119-treated mice. (*E*) Motor performance of motavizumab-treated mice declined over the first week, and mice reached disease-endpoint before the beginning of the second week. ARGX-119 halted the initial decline in grip strength and rotarod performance between 1 and 2 wk.

Treatment with ARGX-119, begun after disease onset, also ameliorated disease caused by a different patient-derived pathogenic antibody, 3B5 (Fig. 5). O_2_ consumption was reduced by >50% in all (8/8) of the motavizumab-treated mice by 2 to 3 wk after 3B5 treatment (5 mg/kg), when the experiment was terminated (Fig. 5 *A*–*C*). In contrast, treatment with ARGX-119, begun after disease onset, halted or attenuated disease progression initiated by 3B5, as assessed by survival, O_2_ consumption, synaptic structure, and motor performance (Fig. 5 *A*–*E*).

Together, these findings indicate that an agonist antibody to MuSK, ARGX-119, can halt or reverse severe symptoms of neuromuscular disease, caused by two different pathogenic antibodies derived from a MuSK MG patient, even when intervention with ARGX-119 began after disease onset in this mouse model of MuSK MG.

### Antibody-Driven MuSK Dimerization.

**Fig. 6.**
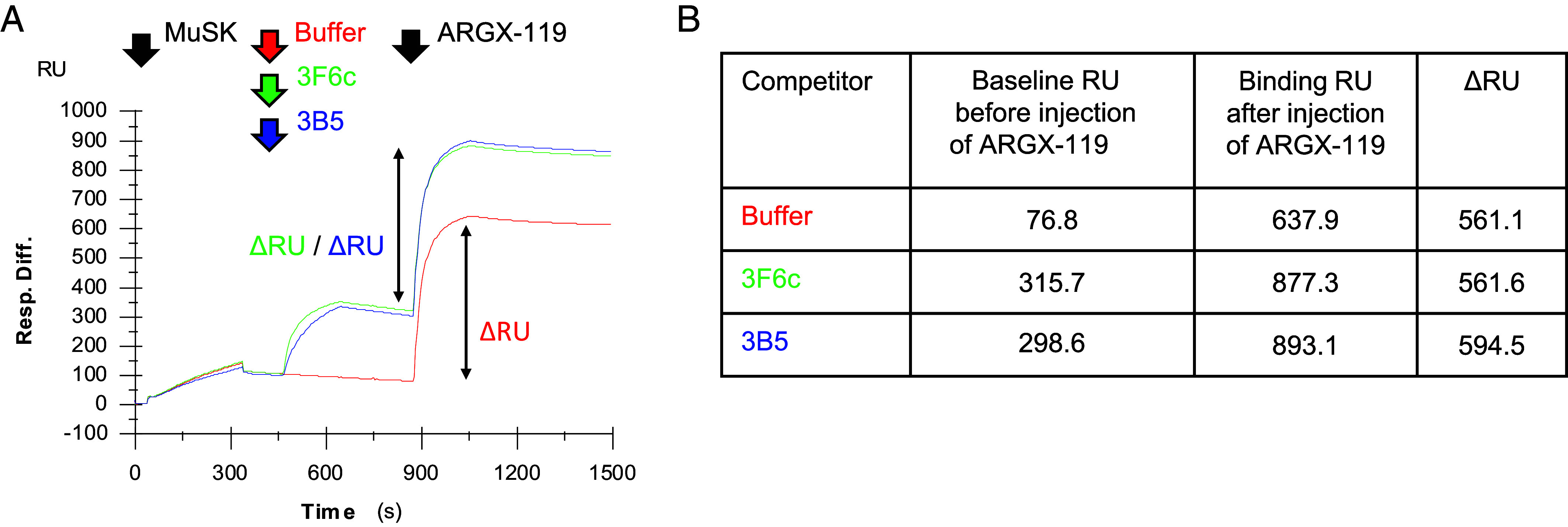
3F6C and 3B5 do not compete with ARGX-119 for binding to mouse MuSK. A monoclonal antibody to Strep-tag II was immobilized to a Biacore CM5 chip by amine coupling. (*A*) The full extracellular region of mouse MuSK, containing a Strep-tag II at the carboxy terminus, was allowed to bind to the monoclonal antibody. Buffer (red), 3F6C (green), or 3B5 (blue), followed by ARGX-119, were passed sequentially over the sensor. 3F6C and 3B5 bind additively with ARGX-119 to the sensor, indicating no competition between ARGX-119 and either 3F6C or 3B5 for binding to MuSK. (*B*) The Table shows that the difference between the RU value after binding of 3F6C or 3B5 and the RU value after binding of ARGX-119 is nearly identical to the RU value for ARGX-119 alone, indicating no competition between 3F6C or 3B5 and ARGX-119.

We considered possible mechanisms by which ARGX-119 might prevent disease in mice treated with 3F6C or 3B5. Although 3F6C and 3B5 bind the first Ig-like domain in MuSK, whereas ARGX-119 binds the MuSK Fz-like domain, we considered the possibility that ARGX-119 might interfere with 3F6C or 3B5 for binding to MuSK, displacing the pathogenic antibodies, thereby restoring MuSK phosphorylation. We used a surface plasmon resonance assay to measure binding between ARGX-119 and MuSK in the presence or absence of 3F6C of 3B5. Fig. 6 shows that ARGX-119 binds to MuSK equally well in the presence or absence of 3F6C or 3B5, indicating that ARGX-119 restores MuSK phosphorylation without competing with 3F6C or 3B5 for binding to MuSK. These data favor the idea that ARGX-119 prevents neuromuscular disease by dimerizing and stimulating MuSK without displacing 3F6C or 3B5 from the first Ig-like domain of MuSK.

## Discussion

Autoimmune diseases are typically managed by generalized immunosuppression, including rituximab to deplete B cells, FcRn blockers to lower circulating antibody levels, inhibitors of complement to reduce cell damage, and steroids that broadly suppress immune function. Despite their benefits, these treatments render patients vulnerable to infection and dampen antibody-mediated surveillance mechanisms. Here, we provide evidence for a therapeutic strategy to treat an autoimmune disease by selectively targeting the disease mechanism.

We show that a single injection of a recombinant, pathogenic antibody to MuSK, derived from a MuSK MG patient, triggers neuromuscular deficits in mice, which resemble neuromuscular disease in MuSK MG patients. Disease in this passive transfer model, which is caused largely if not entirely by inhibition of MuSK phosphorylation, is more severe than MuSK MG in humans, as a single injection of the pathogenic antibodies causes a >50% reduction in O_2_ consumption, requiring sacrifice of the mice within 2 to 3 wk. Despite the severity of this MuSK MG disease model, treatment with a MuSK agonist antibody, ARGX-119, delivered after disease onset, overcomes and reverses disease, providing a proof of principle for treatment of MuSK MG patients.

Prophylactic rescue with ARGX-119 is remarkable, but rescue is even more impressive following ARGX-119 treatment after disease onset. Although these findings may seem counterintuitive, prophylactic treatment with ARGX-119 began when systemic levels of pathogenic antibodies were maximal, whereas postonset treatment with ARGX-119 began when pathogenic antibody levels had declined. Although both experimental paradigms provide a proof-of-principle for this therapeutic strategy, the ability of ARGX-119 to reverse motor decline after disease onset is the more relevant finding from a clinical perspective.

All MuSK-MG patients, described to date, contain IgG4 antibodies that are directed against the first Ig-like domain in MuSK (20, 36). Antibodies 3F6C and 3B5 likewise bind the first Ig-like domain in MuSK and thus represent the most common pathogenic antibodies in MuSK-MG patients. Then, 20 to 30% of MuSK-MG patients additionally carry autoantibodies to other domains in MuSK, notably the second Ig-like domain and the Fz-like domain (20, 36, 37). No MuSK MG patients, described so far, carry antibodies only to the Fz-like domain, suggesting that antibodies to the Fz-like domain are not themselves pathogenic (36). The experiments described here indicate that ARGX-119 has the potential to provide benefit to 70 to 80% of MuSK-MG patients, who carry antibodies only to the first Ig-like domain in MuSK but do provide insight into whether ARGX-119 may provide benefit to the minority of MuSK-MG patients who also carry antibodies to additional domains in MuSK.

Although B cell depletion is an effective strategy for treating MuSK MG, relapse is common, as B cells, which produce antibodies that bind the first Ig-like domain in MuSK, persist and reemerge to disrupt MuSK function (38). As such, ARGX-119 may be a valuable therapeutic for treating MuSK MG patients suffering from relapse after treatment with rituximab as well as newly diagnosed patients.

In MuSK MG, MuSK is the direct target of autoantibodies that interfere with MuSK function and diminish MuSK phosphorylation (15, 20, 21). As such, agonist antibodies to MuSK, such as ARGX-119, appear to be particularly well-suited therapeutics for treating this disease. Stimulating MuSK, either by a MuSK agonist antibody or by *Dok7* overexpression, can provide therapeutic benefit in mouse models of other neuromuscular diseases, including amyotrophic lateral sclerosis, *Dok7* congenital myasthenia, spinal muscular atrophy, and Emery–Dreifuss muscular dystrophy, as well as muscle wasting, sarcopenia, during aging (28, 33, 34, 39–41). The mechanisms by which boosting MuSK phosphorylation ameliorates pathology in mouse models of amyotrophic lateral sclerosis and spinal muscular atrophy are not well understood, as MuSK is neither mutated nor a direct target in these diseases. Presumably, augmenting MuSK phosphorylation boosts poorly understood pathways that act downstream from MuSK to stabilize synapses that would otherwise deteriorate or disassemble, suggesting that ARGX-119 may provide therapeutic benefit for multiple neuromuscular diseases.

## Materials and Methods

Two antibodies, 3B5 and 3F6C, derived from a MuSK MG patient, were engineered as one-armed antibodies with impaired effector function. ARGX-119, a MuSK agonist antibody deficient in effector function, binds selectively to MuSK (29). Mice were monitored continuously in metabolic chambers to record O_2_ consumption, CO_2_ production, and energy expenditure. Motor performance was also assessed by grip strength and Rotarod performance. Synaptic differentiation was assessed by staining for axons, nerve terminals and AChRs in the postsynaptic membrane (see *SI Appendix* for further details).

## Supplementary Material

Appendix 01 (PDF)

## Data Availability

The materials described here may be available for research purposes, subject to a material transfer agreement. Requests for reagents should be sent to Roeland Vanhauwaert (rvanhauwaert@argenx.com) at argenx. All other data are available in the manuscript or *SI Appendix*.
