# Supplementary material for: Agonist antibody to MuSK protects mice from MuSK myasthenia gravis
Source: Proc Natl Acad Sci U S A. 2024 Sep 17;121(39):e2408324121. doi: 10.1073/pnas.2408324121 (PMC11441477; doi:10.1073/pnas.2408324121)
Supplement: Supplementary file 1 — Appendix 01 (PDF) [file pnas.2408324121.sapp.pdf]

**Supporting information**
**Materials and Methods**
**Antibodies**

3B5 and 3F6C were engineered as one-armed antibodies using knobs-into-holes technology (1) and produced on a hIgG1 backbone with mutations (L234A, L235A), which impair effector functions, at Creative Biolabs Biologics (Shirley, NY). Functionally monovalent forms of these antibodies have been described previously and dubbed 11-3F6C and 13-3B5 (2). Here, for simplicity, we describe antibodies 11-3F6C and 13-3B5 as 3F6C and 3B5 (2).

ARGX-119 was isolated from llamas immunized with the Fz-like domain from human MuSK, and the antibody was produced and characterized, as described elsewhere (3). Unlike X17 (4), which binds human and mouse MuSK but cross-reacts with EphB1 and EphB2, we failed to detect binding of ARGX-119 to targets other than MuSK (3). ARGX-119 was engineered on a hIgG1 backbone with mutations (L234A, L235A), which diminished effector function (5), and produced at Lonza Biologics (Slough, UK). The isotype control antibody, termed motavizumab, which targets the Respiratory Syncytial Virus F glycoprotein, was engineered on the same backbone.

**Treatment of cultured myotubes with Agrin, 3F6C and ARGX-119**

C2C12 myotubes were grown in cell culture and treated with neuronal Agrin (550-AG, R&D Systems, Minneapolis, MN), as described previously (4, 6). MuSK was immunoprecipitated from cell lysates with antibody 1A (4), and Western blots were probed with antibodies to MuSK (AF562, R&D Systems, Minneapolis, MN) or phosphotyrosine (4G10, Millipore, Burlington, MA).

**Prophylactic treatment with ARGX-119**

Two- to three-month-old C57BL/6 mice were housed in metabolic chambers and injected intraperitoneally with monovalent 3F6C (5mg/kg) and one day later with either motavizumab (20mg/kg) or ARGX-119 (20mg/kg). Their metabolic functions were monitored continuously.

**ARGX-119-treatment after disease onset**

2- to 3-month-old C57BL/6 mice were injected intraperitoneally with either monovalent 3F6C (7mg/kg) or 3B5 (5mg/kg) and monitored continuously in metabolic chambers to record $O_2$ consumption, $CO_2$ production and energy expenditure. We defined: (1) disease onset as the time when $O_2$ consumption was reduced by 10% on two consecutive days, (b) disease endpoint as the time when $O_2$ consumption was reduced by >50%, and (c) study endpoint as 15 days after treatment with ARGX-119. At disease onset, mice were injected with either motavizumab (20mg/kg) or ARGX-119 (20mg/kg).

**Synaptic differentiation**

Neuromuscular synapses in diaphragm muscles were stained with Alexa 488–$\alpha$-bungarotoxin (BGT) (Invitrogen, Waltham, MA) to label AChRs, and with antibodies to β-III Tubulin (302302, Synaptic Systems, Goettingen, Germany) and Synapsin [106002, Synaptic Systems, Goettingen, Germany) to label axons and nerve terminals, respectively. We analyzed $\geq$50 synapses in each muscle from 3-8 mice in each category, as described previously (4, 7). Synaptic size was defined as the AChR-stained synaptic area.

**Motor performance**

Grip strength and Rotarod performance were determined and analyzed as described previously (4, 7). Respiratory gas utilization was measured by indirect calorimetry using an eight-cage open respirometry system (TSE PhenoMaster, TSE Systems GmbH, Germany), as described previously (8). Mice were weighed and housed individually in Techniplast home cages, located within a temperature- and humidity-controlled climate chamber (22 ± 0.5°C, 50 ± 1% relative humidity). The light cycle was set to 12:12 (lights on at 06:30), and the air flow for each cage was set to 0.35 l/min (0.25 l/min diverted to the gas sensors during the sampling period: 190-s line purge and 10-s active sample). Oxygen consumption [$vO_2$ = ml/hr], carbon dioxide production [$vCO_2$ = ml/hr], and cumulative measures of food consumption [g], water intake [ml] and activity [beam break counts, X+Y+Z axes] were recorded at 30-min intervals. Energy expenditure [EE = kcal/h = $3.941 \times vO_2 + 1.106 \times vCO_2$, ignoring urinary nitrogen] was calculated using Weir's equation (9). Mice were acclimated to the metabolic home cages for at least 24 hours before data were used for analysis. $vO_2$, $vC0_2$ and EE were expressed as percentage of baseline. Data were acquired and exported with TSE PhenoMaster software V8.1.4.14156 (TSE Systems GmbH, Germany, https://www.tse-systems.com/product-details/phenomaster).

Experimenters were blinded from the particular treatment. The study was approved by the IACUC of NYU Medical School under the protocol number of IA16-00080.

**Statistics**

The scatter plots show the individual and mean (± SEM) values. Unless stated otherwise, we used a two-sided Student's *t*-test to determine the statistical significance between different values (ns, not significant; p, *<0.05; p, **<0.005; p, ***<0.0005; p, ****<0.00005), using GraphPad Prism 9.0 software. Because motavizumab treatment failed to rescue 3F6C- or 3B5-treated mice from lethality, statistical comparisons of ARGX-119- and motavizumab-treated mice were only done when three or more motavizumab-treated mice could be included in the analysis.

**Supporting information References**


1. A. M. Merchant *et al.*, An efficient route to human bispecific IgG. *Nat Biotechnol* **16**, 677-681 (1998).
2. D. L. E. Vergoossen *et al.*, Functional monovalency amplifies the pathogenicity of anti-MuSK IgG4 in myasthenia gravis. *Proc Natl Acad Sci U S A* **118** (2021).
3. R. Vanhauwaert *et al.*, ARGX-119, a therapeutic agonist antibody targeting MuSK. *bioRxiv* 10.1101/2024.07.18.604166, 2024.2007.2018.604166 (2024).
4. J. Oury *et al.*, Mechanism of disease and therapeutic rescue of Dok7 congenital myasthenia. *Nature* **595**, 404-408 (2021).
5. M. Hezareh, A. J. Hessell, R. C. Jensen, J. G. van de Winkel, P. W. Parren, Effector function activities of a panel of mutants of a broadly neutralizing antibody against human immunodeficiency virus type 1. *J Virol* **75**, 12161-12168 (2001).
6. R. Herbst, S. J. Burden, The juxtamembrane region of MuSK has a critical role in agrin-mediated signaling. *The EMBO journal* **19**, 67-77 (2000).

7. J. Oury *et al.*, MACF1 links Rapsyn to microtubule-and actin-binding proteins to maintain neuromuscular synapses. *Journal of Cell Biology* **218**, 1686-1705 (2019).
8. M. Voisin *et al.*, Inhibiting LXRalpha phosphorylation in hematopoietic cells reduces inflammation and attenuates atherosclerosis and obesity in mice. *Commun Biol* **4**, 420 (2021).
9. J. B. Weir, New methods for calculating metabolic rate with special reference to protein metabolism. *J Physiol* **109**, 1-9 (1949).
